# Acute and long-term effects of ocular surface burns on ocular surface microbiota: a one-year follow-up study

**DOI:** 10.3389/fcimb.2026.1803003

**Published:** 2026-07-03

**Authors:** Zijing Lu, Shouyue Xia, Na Zhang, Junpeng Liu, Jihong Wang

**Affiliations:** 1 The Affiliated Eye Hospital of Suzhou Vocational Health College, Suzhou, China; 2 Department of Ophthalmology, Affiliated Hospital of Jiangnan University, Wuxi, China; 3 Wuxi School of Medicine, Jiangnan University, Wuxi, China; 4 The First Affiliated Hospital of Guangxi University of Traditional Chinese Medicine, Nanning, China

**Keywords:** 16S rRNA gene sequencing, conjunctival sac secretion, microbial dysbiosis, ocular surface burn, ocular surface microbiota

## Abstract

**Background:**

Ocular surface burns (OSBs) are severe injuries that disrupt the ocular surface microenvironment. While the role of microbiota in ocular surface homeostasis is increasingly recognized, the acute and long-term impacts of OSB on this microbial community remain poorly understood.

**Methods:**

This longitudinal study collected conjunctival swabs from the inferior fornix of three groups: healthy controls (HOS, *N* = 11), patients with acute OSB (*N* = 11), and the same patients one-year post-burn (OSB_1Y, *N* = 7). Microbiota composition was analyzed using 16S rRNA gene amplicon sequencing. Ocular surface parameters and patient-reported outcomes were also assessed.

**Results:**

Exploratory observations suggest trends toward altered ocular surface microbiota in OSB patients that may persist for at least one year. Alpha diversity was lower in OSB and OSB_1Y compared with HOS. At the genus level, *Parabacteroides* and *Lachnoclostridium* showed trends toward enrichment in OSB and OSB_1Y. Possible associations were observed in correlation analyses: in OSB_1Y, *Corynebacterium* abundance tended to be negatively associated with tear meniscus height, while *Bacteroidota* tended to be positively associated with meibomian gland dropout scores. Lower microbial relative abundances were observed with worse visual acuity and more severe burn grade. Predictive functional analyses suggested trends toward upregulation of type III secretion system-related proteins in the acute phase and tight junction—associated proteins in the long-term phase.

**Conclusion:**

OSB may be associated with acute and long-lasting changes in the ocular surface microbiota. Given the low-biomass nature of the ocular surface, the absence of formal contamination controls, the use of a stool-optimized DNA extraction kit, and the lack of multiple testing correction, these findings should be interpreted with extreme caution. These preliminary microbial observations appear nominally associated with some clinical parameters, which may provide exploratory clues for post-burn ocular surface changes, but no definitive pathophysiological relevance can be established.

## 1 Introduction

Ocular surface burns (OSBs) are acute eye emergencies that mostly involve thermal and chemical burns. Accidents such as pyrotechnics, steam, boiling water, or molten metal (usually aluminum) can cause thermal burns. Alkaline or acidic compounds can induce chemical burns (Dua et al., 2001). Sharma et al. reported that chemical burns account for 11.5% to 22.1% of the total number of eye injuries (Sharma et al., 2018). Eye burns, especially OSBs, are more frequent in China than in developed countries, accounting for around 29.9% of hospitalized patients in the country (Duan et al., 2020).

The ocular surface maintains a unique, low-biomass commensal microbiota that is critical for local tissue homeostasis and mucosal defense. Under physiological conditions, the healthy ocular surface microbiota presents a relatively stable, low-diversity composition, dominated by beneficial commensal taxa that inhibit pathogen colonization, regulate local immune responses, and protect epithelial barrier function (Ozkan and Willcox, 2019; Garza et al., 2021; Chiang and Chern, 2022). Recent large-scale, sequencing-based population cohorts in healthy individuals have moved beyond descriptive profiling to formally identify, quantify, and rank host-related, physiological, environmental, and lifestyle factors that independently shape ocular surface microbiota structure and drive interindividual variation. These systematic investigations have defined the magnitude of baseline biological and technical variance, providing essential context for interpreting group-level differences in longitudinal microbiota studies. Key confirmed modifiers include age, sex, environmental exposure, living habits, tear film stability, and ocular surface physiological status, all of which may independently alter microbial community structure and must be considered in study design and interpretation (Wen et al., 2017; Kamdougha et al., 2025; Rizzuto et al., 2025). Beyond these factors, chronic topical pharmacological therapies also independently modify the ocular surface microbiota. Borroni et al. recently reported that long-term use of preserved intraocular pressure-lowering medications in primary open-angle glaucoma patients is associated with significant alterations in ocular surface microbial diversity and community composition compared with healthy controls (Borroni et al., 2026). The structure and dynamic balance of ocular surface microbial communities are thus continuously shaped by multiple internal and external confounding factors, including tear film stability, mucosal integrity, daily environmental exposure, living habits, and long-term topical medication intervention. Accumulating clinical evidence has confirmed that chronic ocular diseases and prolonged topical drug treatment can significantly disrupt physiological microbial distribution, reduce community diversity, deplete beneficial symbiotic bacteria, and enrich conditional pathogenic microbiota, ultimately driving persistent ocular surface microenvironmental dysregulation (Aguilera et al., 2020; Shao et al., 2023). The ocular surface represents an extremely low-biomass environment, which presents unique methodological challenges, including high susceptibility to environmental and reagent contamination. Distinguishing authentic ocular taxa from gut-associated or kit-derived contaminants is critical for reliable interpretation. Furthermore, ocular burns trigger complex local inflammation, tear film disruption, and prolonged clinical interventions that may independently confound microbial composition.

Recent studies have shown widespread involvement of the microbiota in multiple acute and chronic eye diseases (Janowitz et al., 2019; Deng et al., 2021; Huang et al., 2023). Pathological damage to the ocular surface can reshape microbial composition, weaken the original protective effect of commensal flora, and further aggravate local inflammation and tissue repair disorders. In addition, mood agitation and sleep disturbance are highly prevalent among OSB patients, commonly triggered by severe ocular pain and long-term psychological anxiety regarding visual impairment after injury (Guerrero-Moreno et al., 2020). Nevertheless, longitudinal changes in the ocular surface microbiota following combined chemical and thermal burn injury, as well as the correlation between microbial dysbiosis, clinical manifestations, and psychological scale indicators, remain poorly clarified.

Therefore, the primary aim of the current study was to investigate the acute and long-term longitudinal alterations of the ocular surface microbiota after OSB and to further explore the potential associations between key clinical parameters, functional pathway changes, and microbial community characteristics.

## 2 Materials and methods

### 2.1 Subjects

This study was approved by the Medical Ethics Committee of Jiangnan University (JNU20220310IRB46) and conducted in accordance with the Declaration of Helsinki. Patients with ocular surface burn (OSB, *N* = 11) and healthy persons (HOS, *N* = 11) were recruited at Jiangnan University’s Affiliated Hospital from January to December 2024. Among the 11 patients with OSBs, seven completed the one-year follow-up. The demography was shown in **Supplementary Table 1**. Group comparisons (HOS vs. OSB vs. OSB_1Y) included all 11 HOS, all 11 OSB, and all seven OSB_1Y samples. Paired within−subject comparisons (OSB vs. OSB_1Y) were restricted to the seven matched patients with complete follow-up data. Written consent was obtained from each participating subject after explanation of the nature of the study. The inclusion and exclusion criteria for OSB and HOS were as follows. For the OSB group, the inclusion criterion was being diagnosed by an ophthalmologist to have OSB without other burns on the body; exclusion criteria were the presence of systemic, local, eyelid, corneal, conjunctival, or other infectious diseases based on medical history and slit lamp examination; history of diabetes or other conditions affecting the ocular surface; and history of ocular trauma or surgery. For the HOS group, the inclusion criterion was no OSB, as confirmed by slit lamp examination that excluded infectious diseases of the eyelid, cornea, and conjunctiva; exclusion criteria were history of diabetes or other diseases that could affect the ocular surface, history of ocular trauma or surgery, and history of contact lens wearing. Healthy controls were enrolled with comparable age (mean ± SD: 56 vs. 45 years, *p* = 0.193) and sex distribution (male: 81.8% vs. 100%, *p* = 0.478) to the OSB group. All participants were recruited from the same geographic region and clinical setting to minimize environmental variability.

### 2.2 Sample and questionnaire collection

**Figure 1:**
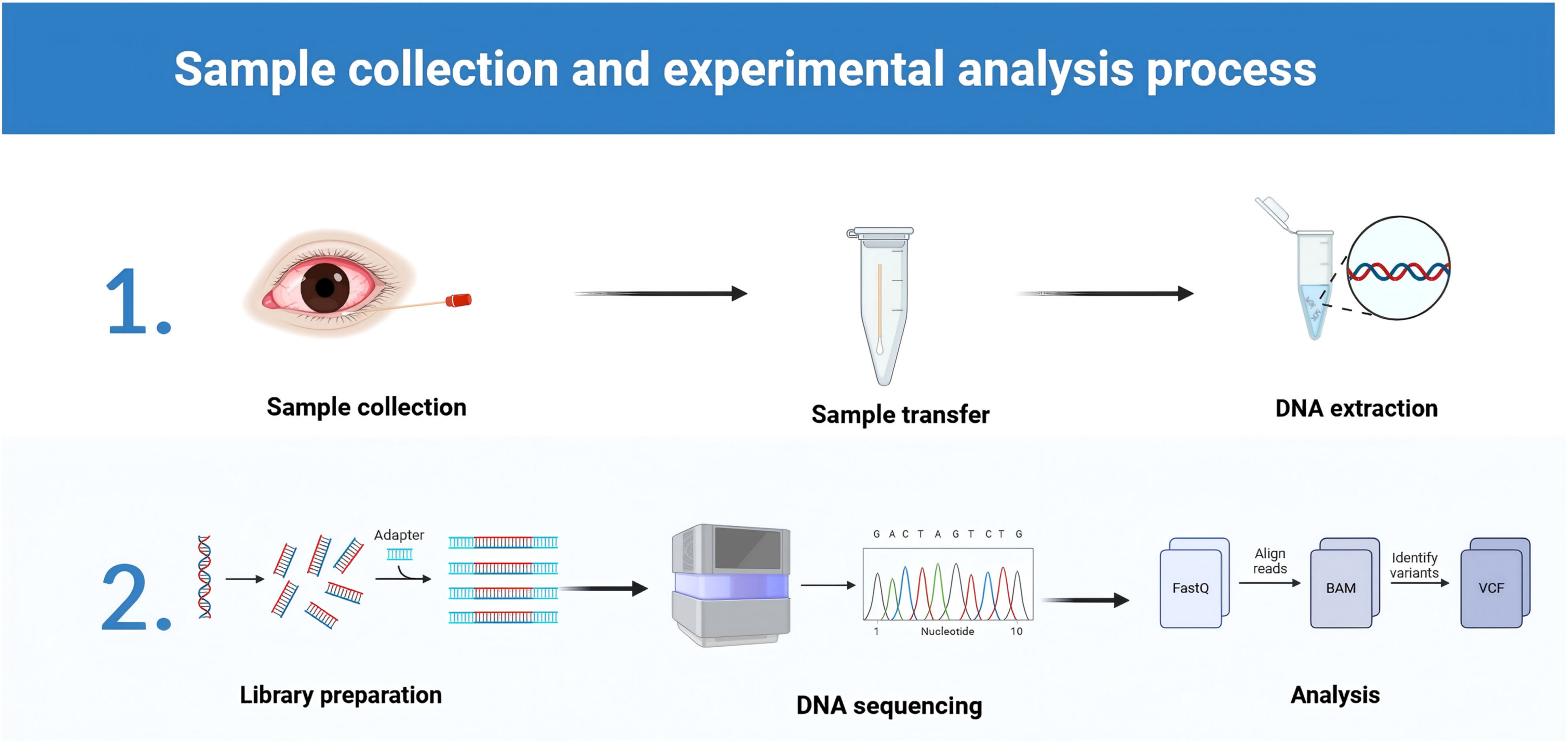
Sample collection and experimental analysis process.

Conjunctival sac secretions were collected from the OSB (patients with ocular surface burn), OSB_1Y (one year after OSB), and HOS (healthy persons) groups. On admission, patients with OSB received only emergency treatments, such as quick irrigation of the eyes. Before the patient used any antibiotics or eye drops, a sample was collected from the conjunctival sac of one eye (**Figure 1**). Each participant selected one eye for the test; for patients with unilateral burns, the affected eye was chosen; for patients with bilateral burns or healthy controls, the right eye was selected for the test. Before sampling, the patient was asked to look upward, pull back the lower eyelid, expose the lower bulbar conjunctiva and lower fornix conjunctiva, and use a sterile disposable sampling swab to swab the lower conjunctival sac and lower eyelid conjunctival surface from the inner canthus and rotate from inside to outside, avoiding contact with eyelashes and eyelid margin. Conjunctival sac pH was not measured in this study. Although all OSB patients received emergency ocular irrigation upon admission, residual pH abnormalities may still have been present and could influence the ocular surface microenvironment and microbial composition. Patients with OSB_1Y were instructed to have a return visit in the morning and not to use any medication until the return visit.

Following the sample, antibiotics or eye drops for decreasing eye pressure and dilating eye drops were given as needed. Samples were collected by using sterile transport swabs (Zhejiang Medical Device Co., Ltd., Hangzhou, China) without touch of eyelash or eyelids during sampling. Each collected swab was placed in a sterile tube and stored at −80°C immediately until DNA extraction.

The McGill Pain Questionnaire (SF-MPQ) (Yamanishi et al., 2019) and Pittsburgh Sleep Quality Index (PSQI) (Wang and Wang, 2026) were collected from patients with OSB (OSB) before discharge and one year later (OSB_1Y). The SF−MPQ was selected because it provided a well−validated, multidimensional assessment of both sensory and affective pain dimensions with low respondent burden, whereas the PSQI was chosen as the reference standard for global sleep quality assessment. The McGill Pain Questionnaire assessed pain sensation and emotion and was widely regarded as a gold standard of pain measurement instruments and consisted of four grades: none, mild, moderate, and severe (Yoshikawa et al., 2021). The Pittsburgh Sleep Quality Index was used to evaluate the participants’ sleep quality during the previous month. The total score ranged from 0 to 21, with higher numbers indicating lower sleep quality (Buysse et al., 1989). When filling out the questionnaire, all participants were ensured to clearly comprehend all of the items.

Treatment exposure during the one-year follow-up period was recorded via medical chart review for all patients in the OSB_1Y group. Standard clinical care included topical antibiotics, corticosteroids, preservative-containing lubricants, cycloplegics, and intraocular pressure-lowering agents as clinically indicated. Several patients received additional interventions such as amniotic membrane transplantation for severe ocular surface damage. A detailed summary of treatments received is provided in **Supplementary Table 2**. While all patients discontinued topical medications on the morning of follow-up sampling, long-term treatment exposure represents a potential confounding factor that may influence ocular surface microbiota composition.

### 2.3 Ocular surface parameter assessment

To obtain the ocular surface parameter, Keratograp 3 (Oculus, Wetzlar, Germany) was used to scan the ocular surface of all the participants in the OSB_1Y group, and four ocular surface parameters obtained were as follows: tear meniscus height (TMH), tear breakup time (BUT), Schirmer tear test (STT) (Singh et al., 2023), and the meibomian gland dropout score (MGDS) (Srinivasan et al., 2012). TMH was the distance between the darker edge of the lower eyelid and the upper limit of the tear meniscus. BUT was categorized as moderate (5 to 10 s) or severe (less than 5 s) (Singh et al., 2019; Wang et al., 2023). The Schirmer tear test (STT) was conducted as the basic tear secretion test without topical anesthesia, using filter paper strips (Tianjin Jingming New Technological Development Co., Ltd., China) following the manufacturer’s instructions. The results measured by the length of the wetted strip were interpreted as follows: hyposecretion (< 5 mm) and severe hyposecretion (5–10 mm). MGDS were divided into four grades based on the grading scale as previously described in Arita’ s report (Arita et al., 2010): Grade I with no area loss, grade II with loss area less than 33%, grade III with loss area between 33% and 67%, and grade IV with loss area more than 67%. The grade of OSB was based on Dua (Dua et al., 2001). Visual impairment was graded according to the national standard GB-11533-2011: Normal vision: BCVA ≥ 0.3; Level IIlow vision: 0.1 ≤ BCVA < 0.3; Level II blindness: 0.02 ≤ BCVA < 0.05 or visual field radius < 10°; Level I blindness: BCVA < 0.02 or visual field radius < 5. BCVA, best-corrected visual acuity.

### 2.4 DNA extraction and 16S rRNA gene sequencing

Microbial DNA was extracted using the TIANamp Stool DNA kit (Catalog No. DP328, Tiangen, Beijing, China). The hypervariable V4 regions of bacterial 16S rRNA genes were amplified by polymerase chain reaction using V4-specific primers 515F (5’-GTGCCAGCMGCCGCGGTAA-3’) and 806R (5’-GGACTACHVGGGTWTCTAAT-3’). PCR products were verified using 2% agarose gel electrophoresis, purified using the Gene JET Gel Extraction Kit (Catalog No. K0691), and sequenced on an Ion S5XL sequencer (both from Thermo Fisher Scientific, Waltham, MA, USA), and sequenced on an Ion S5XL sequencer (Thermo Fisher Scientific) with single-end 400-bp read configuration. The average input sequencing depth was 52,816 reads per sample. After quality filtering, denoising, and chimera removal, the mean number of non-chimeric reads was 24,712 reads per sample, with a minimum of 3,447 non-chimeric reads across all samples. All samples were retained for downstream analysis with no exclusions due to insufficient sequencing depth.

### 2.5 16S rRNA gene sequence analysis

The 16S rRNA gene sequencing reads were analyzed using the QIIME 2 pipeline (version 2020.11.0; https://qiime2.org) (Bolyen et al., 2019). Low-quality and chimeric sequences were filtered using the DADA2 program (Callahan et al., 2016). Amplicon sequence variants were generated at 100% sequence similarity. Taxonomy assignment was performed using the q2-feature-classifier against the SILVA 16S rRNA gene database (release r132) with a 99% similarity threshold (Quast et al., 2013). To ensure comparability across samples, all samples were rarefied to the minimum non-chimeric read depth of 3,447 reads for alpha-diversity, beta-diversity, and taxonomic composition analyses. Relative abundance normalization was used for differential abundance testing and correlation analysis. Functional profiles of the microbial community were predicted from 16S rRNA gene data using PICRUSt2 (https://github.com/picrust/picrust2).

### 2.6 SparCC network analysis

To characterize microbial co-occurrence patterns, SparCC (Sparse Correlations for Compositional data; https://bitbucket.org/yonatanf/sparcc) was used to estimate correlation networks at the genus level. Genera with mean relative abundance <0.01% or present in <20% of samples within a group were removed before analysis. SparCC was run with 100 iterations to compute correlation coefficients and pseudo *p*-values. A correlation was considered nominal if the absolute correlation coefficient >0.3 and *p* < 0.01. SparCC was used for co-occurrence network analysis (Friedman and Alm, 2012). Network visualization was performed using Cytoscape (https://cytoscape.org) (Shannon et al., 2003).

### 2.7 Statistical analysis

Continuous variables were expressed as mean ± standard deviation or median (interquartile range). Statistical tests were applied as follows: comparisons among three groups (HOS vs. OSB vs. OSB_1Y) were performed using the Kruskal–Wallis test with Dunn’s *post-hoc* correction for multiple comparisons (Dunn, 1964). Independent two-group comparisons were performed using the Mann–Whitney *U* test. Paired within-subject comparisons between OSB and OSB_1Y, based on 7 matched patients, were analyzed using the Wilcoxon signed-rank test. Beta-diversity differences were tested using PERMANOVA. Correlation analyses were performed using Spearman rank correlation. All tests were two-sided, and *p* < 0.05 was considered statistically notable for descriptive purposes. All results are exploratory and unadjusted for multiple comparisons, consistent with a hypothesis-generating study. Given the large number of comparisons across diversity metrics, taxonomic levels, correlation analyses, network construction, and functional prediction, no adjustment was made for multiple testing. All statistical results are exploratory and hypothesis-generating only. Nominal *P*-values are reported for descriptive purposes, and findings should not be interpreted as confirmatory. Statistical analyses were performed using GraphPad Prism V.9.0 (GraphPad Software, San Diego, CA, USA).

## 3 Results

### 3.1 OSB led to decreased diversity and richness in the ocular surface microbiota

**Figure 2:**
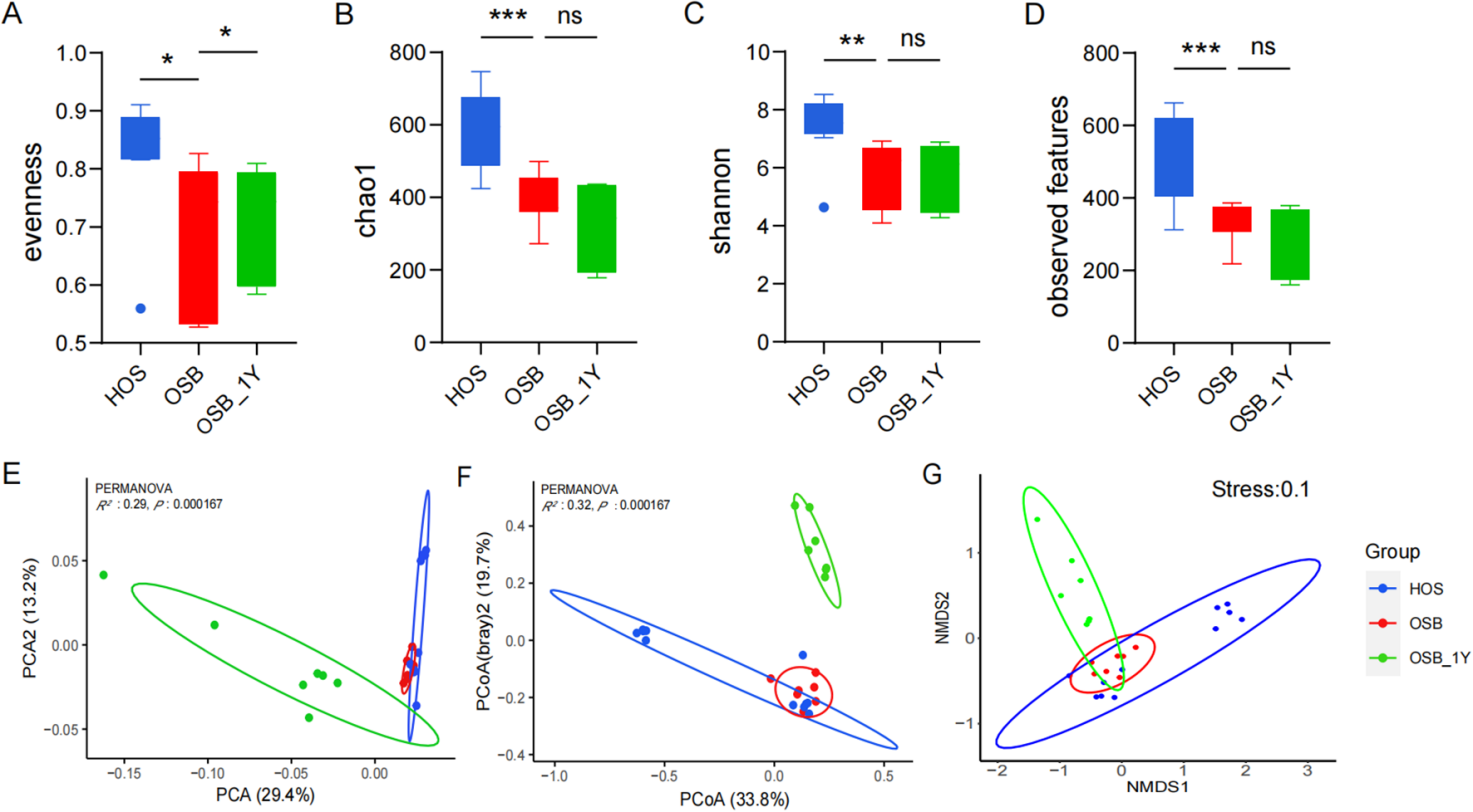
Comparisons of the alpha-diversity and beta-diversity among three groups. Evenness index **(A)**, Chao1 index **(B)**, Shannon index **(C)**, and observed features index **(D)** for species richness and evenness. Three-group comparisons were performed using the Kruskal–Wallis test with Dunn’s *post-hoc* correction. Paired comparison was performed using the Wilcoxon signed-rank test. **(E)** PCA shows similarities and differences between groups based on sample distances. **(F)** PCoA plot based on the Bray–Curtis distance matrix of all samples. **(G)** NMDS showing the distribution of all samples. Group differences in beta-diversity were tested by PERMANOVA. Nominal *P*-values are shown for descriptive purposes only. *nominal *P* < 0.05, **nominal *P* < 0.01, ***nominal *P* < 0.001. (HOS *n* = 11, OSB *n* = 11, OSB_1Y *n* = 7).

To evaluate the effects of OSB on the ocular surface microbiota, we first analyzed alpha diversity and community composition (**Figures 2A–D**). The alpha diversity indices (Chao1 and observed species) of the OSB and OSB_1Y groups showed nominal reductions relative to the HOS group (*P* < 0.05; Kruskal–Wallis with Dunn’s *post-hoc*), consistent with observed trends toward disrupted microbial community structure following OSB. Specifically, the Chao1 index and observed species index in OSB and OSB_1Y were nominally lower than in HOS, consistent with observed trends toward reduced microbial diversity after ocular surface injury. PCA, PCoA, and NMDS confirmed differences in beta diversity among the three groups (**Figures 2E–G**; PERMANOVA, *P* < 0.001). The OSB_1Y group appeared to cluster separately from HOS and OSB, consistent with trends toward persistent microbial alterations. These nominal observations may suggest that OSB are associated with trends toward persistent alterations in the ocular surface microbiota, rather than transient changes.

### 3.2 Changes of microbiota composition in OSB

**Figure 3:**
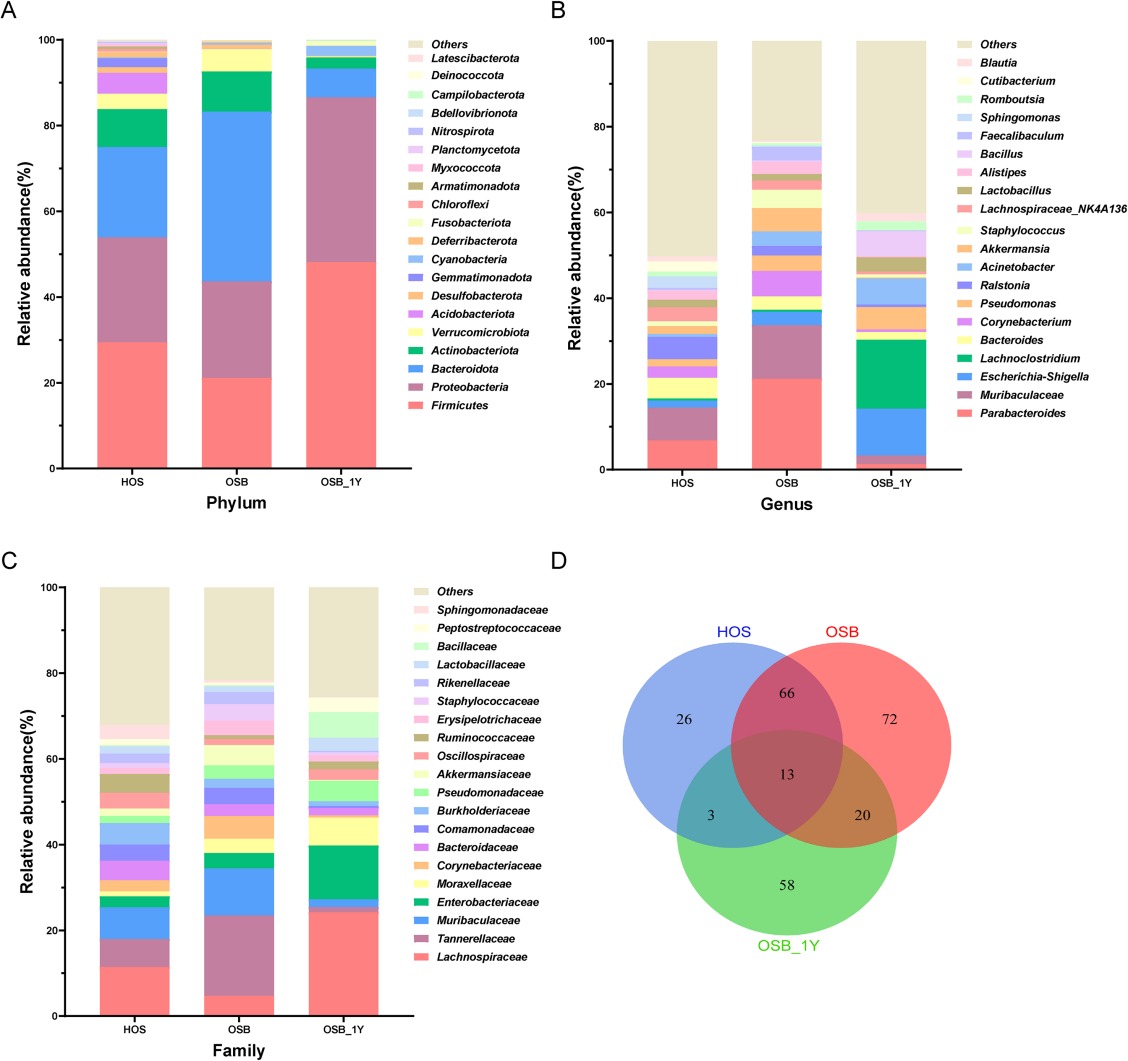
Differences in relative mean abundances of microbiota among three groups. Each phylotype (1% of average relative abundance in groups) is indicated by a different color at the phylum **(A)**, the genus **(B)**, and family **(C)** levels. **(D)** Venn plot illustrating overlap of ocular surface microbial genera among groups. *P < 0.05, **P < 0.01, ***P < 0.001.

**Figure 4:**
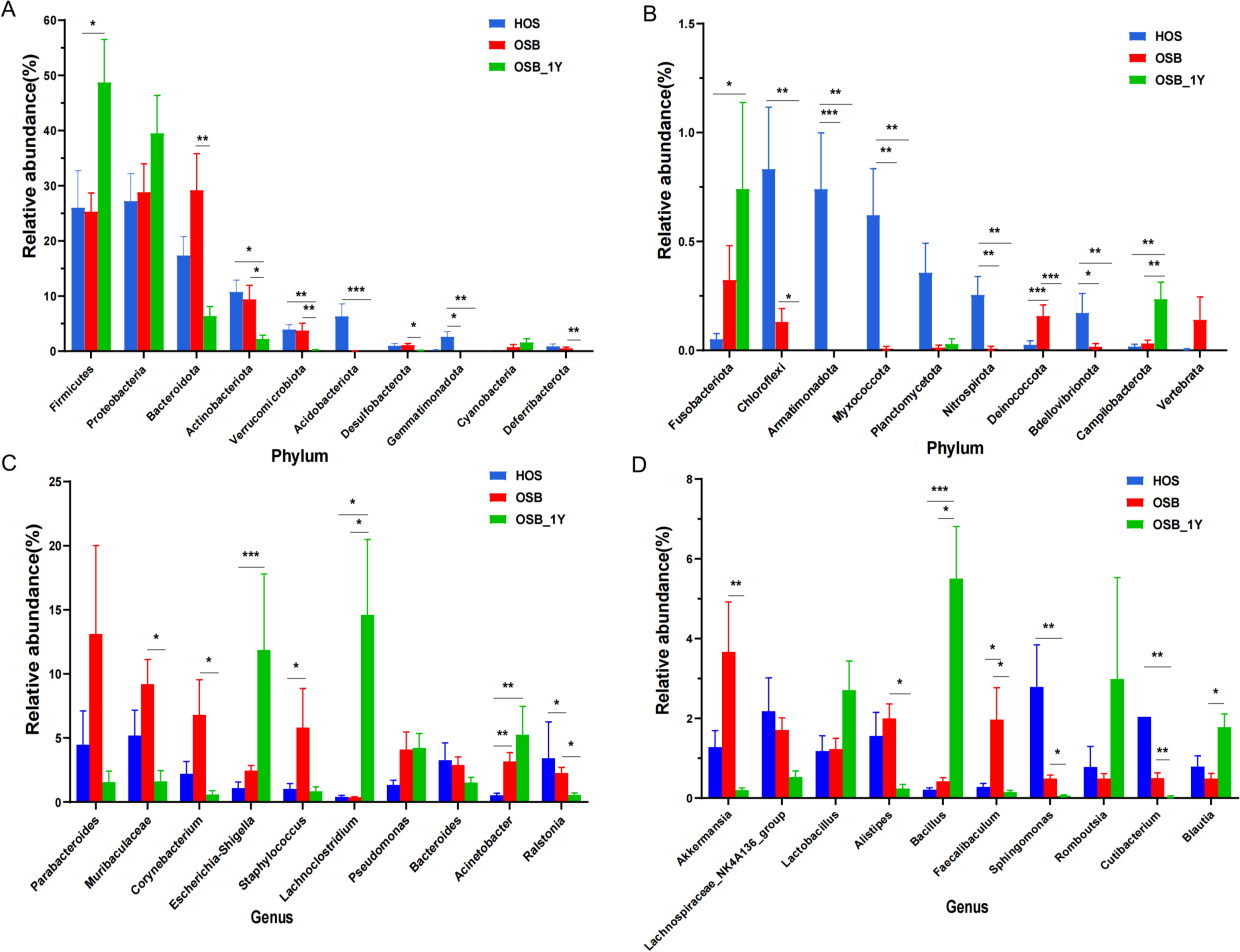
The relative abundances of dominant phylum and genus among three groups. The top twenty ocular surface microbiota in regard to relative abundance at the phylum **(A, B)** and genus **(C, D)** level. *P < 0.05, **P < 0.01, ***P < 0.001.

The relative abundance of microbiota on the ocular surface was then analyzed among the three groups. The distribution of microbiota at different levels was shown in **Figures 3A–C**. The Venn diagram depicts the distribution of the three groups’ basic microbiota at the genus level (**Figure 3D**). We used LefSe to determine the differential microbiota with the richest abundances at the genus level in the three groups (**Supplementary Figure 1**). At the phylum level, four of the top 5 dominant microbiota in each group were shared among the three groups, including *Firmicutes*, *Bacteroidota*, *Proteobacteria*, and *Actinobacteriota*. However, the order ranked by relative abundance was different among them. *Proteobacteria* was the most abundant in HOS, and *Bacteroidota* and *Firmicutes* were the most abundant in OSB and OSB_1Y, respectively (**Figures 4A, B**). Furthermore, most phyla exhibited a decreasing tendency in OSB and OSB_1Y, but *Verrucomicroiota*, *Bacteroidiota*, *Deinococcota*, and *Fusobacteriota* showed an increasing tendency.

At the genus level, *Muribaculaceae* had the highest abundance in the HOS group (**Figures 4C, D**). *Parabacteroides* showed observed trends toward enrichment in the OSB group; however, this taxon is recognized as a potential gut-associated or kit-derived contaminant in low-biomass ocular samples and cannot be interpreted as an authentic ocular resident. *Muribaculaceae*, *Parabacteroides*, *Rolstonia*, *Bacteroides*, and *Lachnoclostridium* were the top five dominant microbiota in the HOS group. The major microbiota in the OSB group were *Parabacteroides*, *Muribaculaceae*, *Corynebacterium*, *Akkermansia*, and *Staphylococcus*. The dominant microbiota in the OSB_1Y group were *Lachnoclostridium*, *Escherichia-Shigella*, *Acinetobacter*, *Bacillus*, and *Pseudomonas*. With the exception of *Corynebacterium*, *Acinetobacter*, *Akkermansia*, *Muribaculaceae*, *Parabacteroides*, *Faecalibaculum*, and *Alitipes*, most microbial abundance decreased after the burn.

*Lachnospiraceae* was reported in the most abundance at the family level in the HOS group and OSB_1Y. The *Tannerellaceae* has the highest abundance in the OSB group (**Supplementary Figures 2A, B**). The top 5 dominating microbiota in the OSB group were *Tannerellaceae*, *Muribaculaceae*, *Corynebacteriaceae*, *Lachnospiraceae*, and *Akkermansiaceae*. Surprisingly, *Lachnospiraceae* was one of the top 5 dominating microbiota in all three groups.

### 3.3 Correlation analysis between clinical parameters and microbiota in OSB and OSB_1Y

**Figure 5:**
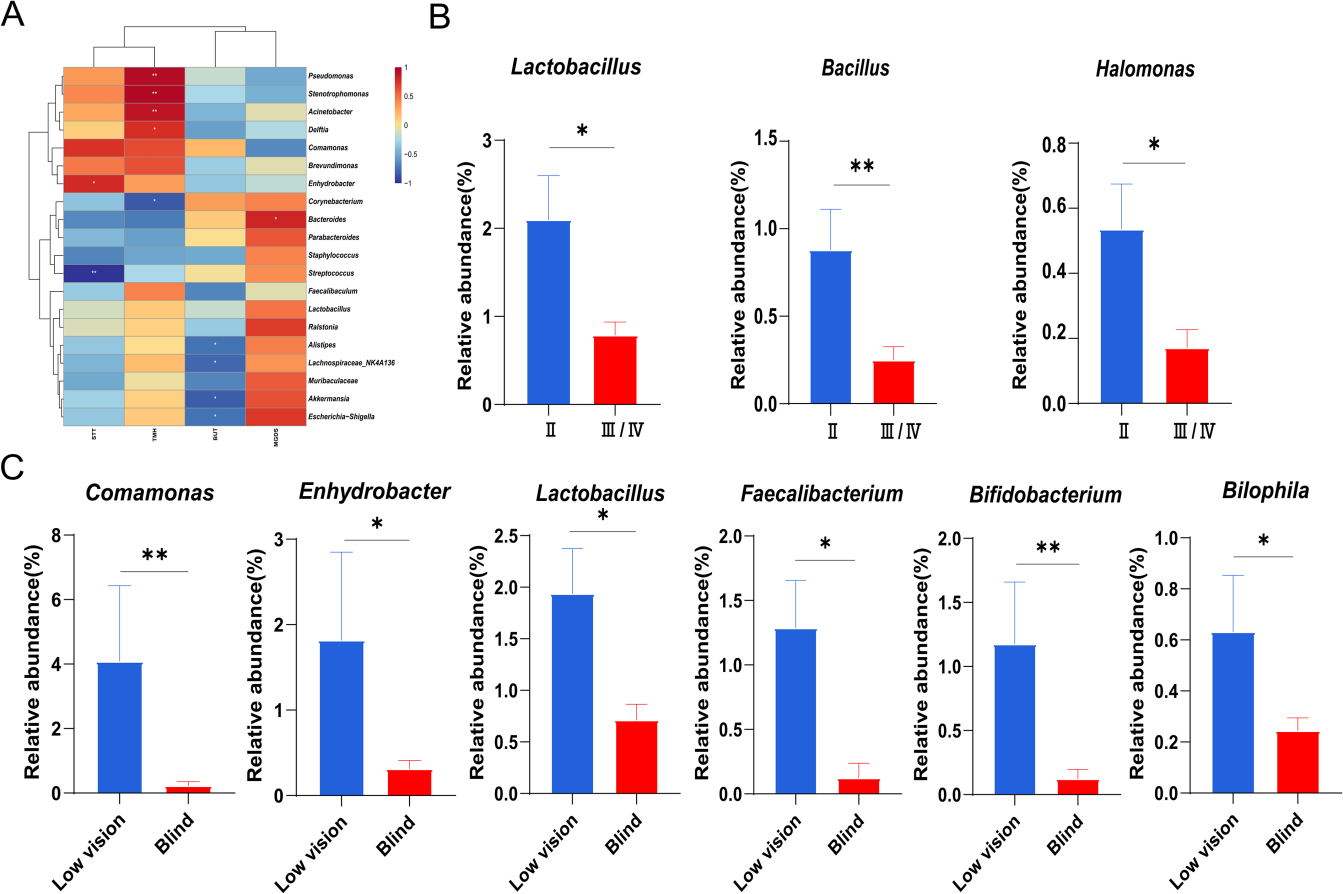
Correlation analysis between clinical parameters and microbiota in OSB and OSB_1Y. **(A)** The top thirty ocular surface microbiota with higher Spearman’ s correlation coefficients with STT, TMH, BUT and MGDS are shown at the genus level in the OSB_1Y group. **(B, C)** There were nominal differences in the average relative abundance of some microbiota according to burn grade and vision result in the OSB group. TMH, tear meniscus height; BUT, the tear breakup time, STT, the Schirmer tear test; MGDS, meibomian gland dropout score. Ocular surface burn grade is based on Dua’ s study.

Spearman rank correlation analyses were performed to explore potential associations between the relative abundances of the top 30 genera and ocular surface parameters (BUT, TMH, STT, MGDS) (**Figure 5A**). These analyses were exploratory in nature and intended to generate hypotheses for future investigation. Several genera presented correlation patterns of potential research value. *Pseudomonas*, *Stenotrophomonas*, *Acinetobacter*, and *Delftia* displayed positive correlation coefficients with TMH, whereas *Corynebacterium* showed a negative correlation coefficient with TMH. *Alistipes*, *Lachnoclostridium*, *Akkermansia*, and *Escherichia-Shigella* exhibited negative correlation coefficients with BUT. *Staphylococcus* showed a negative correlation coefficient with STT, while several hydrophilic genera tended to be positively correlated with STT. *Bacteroides* displayed a positive correlation coefficient with MGDS. Regarding burn grade (**Figure 5B**), *Lactobacillus*, *Bacillus*, and *Halomonas* were the genera that showed the most pronounced correlation patterns. The severity of burns showed a nominal inverse trend with the relative abundance of these three microbiota. This observation is taxon-specific and should not be extrapolated to the microbiota as a whole. Finally, correlations between visual acuity and genus-level relative abundances were explored (**Figure 5C**). Six genera showed patterns consistent with the burn-grade analysis, where lower visual acuity tended to accompany reduced relative abundances of these specific taxa.

### 3.4 Differences in scale scores after OSB

**Figure 6:**
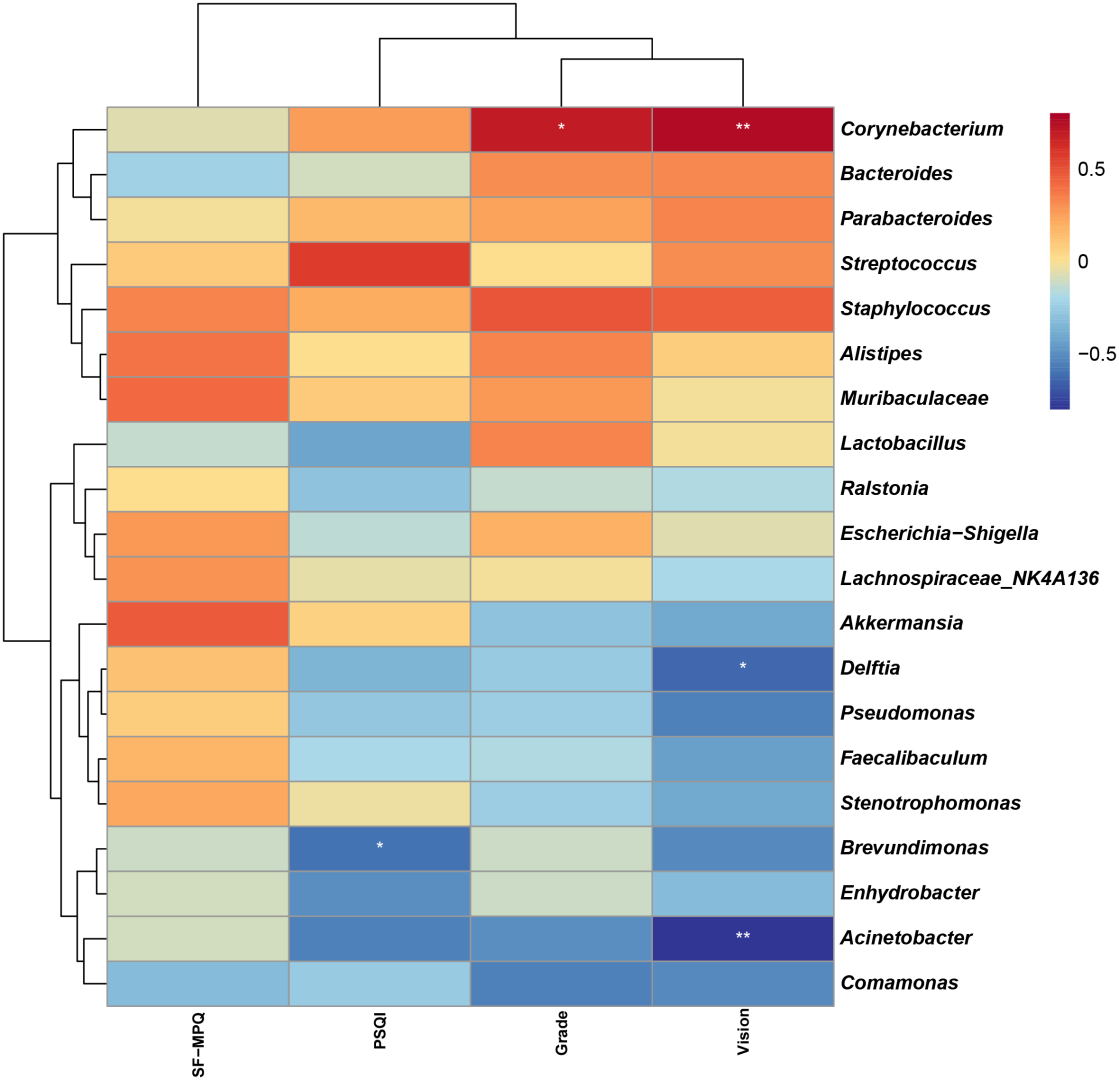
Differences in scale scores after OSB. PSQI, the Pittsburgh Sleep Quality Index; SF-MPQ, the Mc-Gill Pain Questionnaire. Grade, ocular surface burn grade is based on Dua’s study. The low vision assessment was based on the national standard (GB-11533-2011).

The early pain of OSB is obvious, and patients are susceptible to emotional agitation as a result of the worry of lifelong blindness, which causes sleep disorders. At discharge and one year after discharge, we assessed patients’ pain and sleep status using the McGill Pain Questionnaire (SF-MPQ) and the Pittsburgh Sleep Quality Index (PSQI). As can be seen from **Table 1**, the OSB group tended to have poorer overall sleep quality compared with the HOS group. Then, we analyzed the relationship between the patients’ ocular surface microbiota and their vision, burn degree, and the aforementioned two questionnaires in the OSB group (**Figure 6**). *Corynebacterium* showed nominal trends with visual acuity and burn grade. *Enhydrobacter* exhibited a nominal trend toward a negative association with sleep scores, and *Acinetobacter* tended to correlate negatively with visual acuity.

**Table 1:** Comparison of sleep and pain scores between different groups.

	HOS		OSB		OSB_1Y	
	*n* = 11	*p*-value (HOS vs. OSB_1Y)	*n* = 11	*p*-value (HOS vs. OSB)	*n* = 7	*p*-value (OSB vs. OSB_1Y)
SF-MPQ score						
PRI	2.36 ± 1.50	***p* < 0.0001**	13.09 ± 2.95	***p* < 0.0001**	7.91 ± 2.63	**p < 0.05**
VAS	0	***p* < 0.0001**	4.00 ± 1.10	***p* < 0.0001**	2.09 ± 1.30	**p < 0.05**
PPI	0	**p < 0.05**	2.27 ± 0.91	***p* < 0.0001**	1.36 ± 0.92	**p < 0.05**
Total	5.36 ± 1.50	***p* < 0.0001**	19.36 ± 4.57	***p* < 0.0001**	11.36 ± 4.0	**p < 0.05**
PSQI score						
SQ	1.18 ± 0.98	0.898	2.00 ± 0.63	**p < 0.05**	1.09 ± 0.83	**p < 0.05**
SL	2.18 ± 1.66	0.562	2.00 ± 0.78	0.949	1.45 ± 0.52	0.116
ST	1.45 ± 0.69	0.519	2.09 ± 0.70	0.056	1.18 ± 0.60	**p < 0.05**
SE	0.82 ± 0.75	0.949	1.45 ± 0.52	0.065	0.82 ± 0.60	**p < 0.05**
SD	1.18 ± 0.98	1.000	2.18 ± 0.60	**p < 0.05**	1.18 ± 0.87	**p < 0.05**
Hypnotic	0.64 ± 0.81	0.898	1.18 ± 0.98	0.217	0.55 ± 0.69	0.133
DD	0.55 ± 0.52	0.365	1.64 ± 0.67	**p < 0.05**	0.82 ± 0.60	**p < 0.05**
Total	8.00 ± 2.05	0.438	12.55 ± 2.70	***p* < 0.0001**	7.09 ± 3.42	**p < 0.05**

HOS, healthy ocular surface; OSB, ocular surface burn; OSB_1Y, one year after OSB. PSQI, the Pittsburgh Sleep Quality Index; PRI, pain rating index; VAS, visual analog scoring; PPI, present pain level; SF-MPQ, the McGill Pain Questionnaire; SQ, sleep quality; SL, sleep length; ST, sleep time; SE, sleep efficiency; SD, sleep disorders; DD, daytime dysfunction; Data are presented as mean ± standard deviation. HOS vs. OSB and HOS vs. OSB_1Y were analyzed using the Mann–Whitney *U* test. OSB versus OSB_1Y (paired samples) were analyzed using the Wilcoxon signed-rank test. *P* < 0.05 is reported as nominal for descriptive purposes only.

The bold values indicate statistical significance (P < 0.05).

### 3.5 Ocular surface microbiota networks and their key driver genera

**Figure 7:**
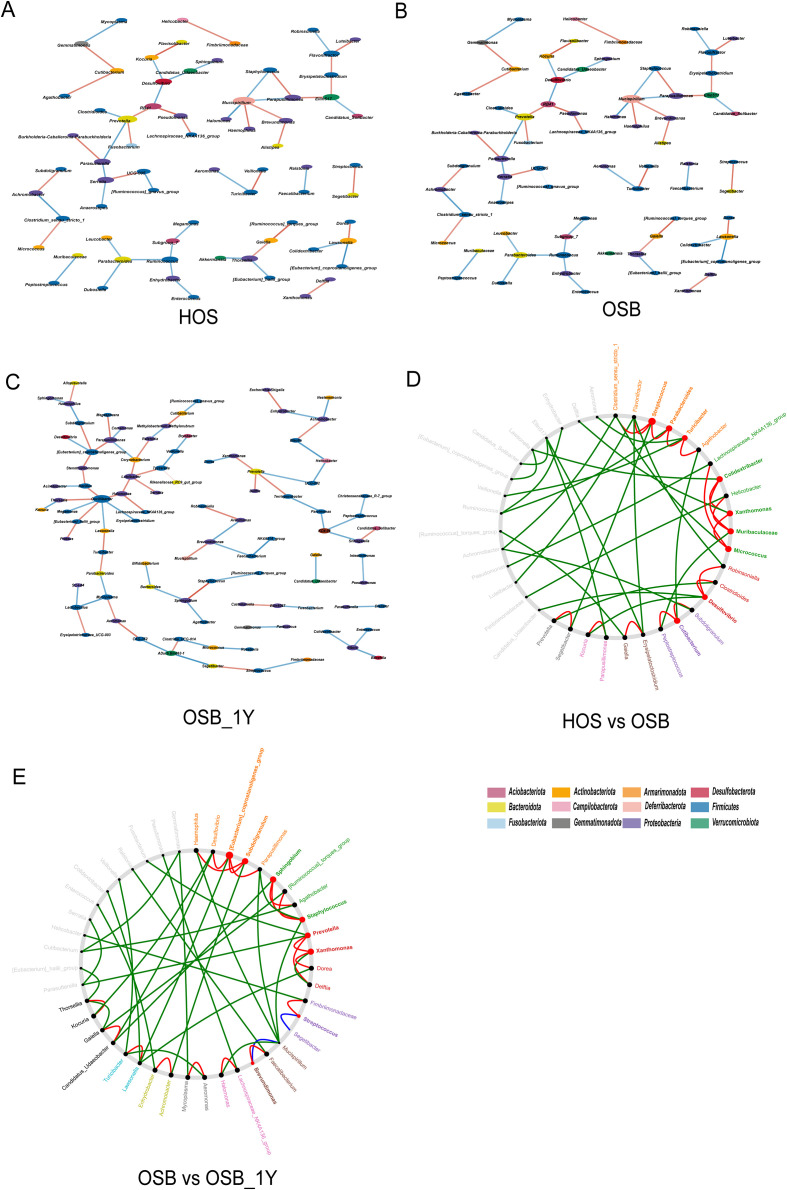
The interactive networks of ocular surface microbiota. Microbial interactive networks in the HOS **(A)**, OSB **(B)**, and OSB_1Y group **(C)** are constructed from SparCC results. NetShift **(D, E)** common sub-networks based on the SparCC networks with highlighted driver genera. Node colors denote the phylum of the genera. Node sizes are in proportion to their NESH scores, and potential drivers are highlighted red. Edges present only in case are colored red, green only in control, and blue in both. SparCC, sparse compositional correlation; NESH, neighbor shift.

Then, we further used the sparse compositional correlation (SparCC) to explore the interaction among microbiota in the three groups. The networks for the bacterial community in the different groups are shown in **Figure 7**. *Muribaculaceae*, the most enriched genus in the HOS group, tended to correlate negatively with *Helicobacter* and *Colidextribacter* (**Figure 7A**). And positively correlated with *Roseburia* and *Oscillibacter*. Furthermore, *Parabacteroides* was found to be negatively correlated with *Leucobacter*, *Ruminococcus*, and *Dubosiella* in the OSB group (**Figure 7B**). Interestingly, *Acinetobacter* and *Bacillus* were the dominant members in the OSB_1Y group, and they were negatively correlated with each other (**Figure 7C**). Moreover, NetShift analysis was used to identify network rewiring and potential key microbial contributors to community shifts (**Figures 7D, E**). Unexpectedly, *Parabacteroides*, *Streptococcus*, *Muribaculaceae*, and *Desulfovibrio* were found to be driver genera responsible for the microbial changes between the HOS group and the OSB group, though *Parabacteroides* and *Muribaculaceae* may represent contaminants. The *Eubacterium coprostannoligenes* group was the particularly important driver. *Streptococcus*, *Mucisirillium*, *Segetibacter*, and *Brevundimonsa* were involved in the change of ocular surface microbiota between the OSB group and the OSB_1Y group.

### 3.6 Functional changes of ocular surface microbiota

**Figure 8:**
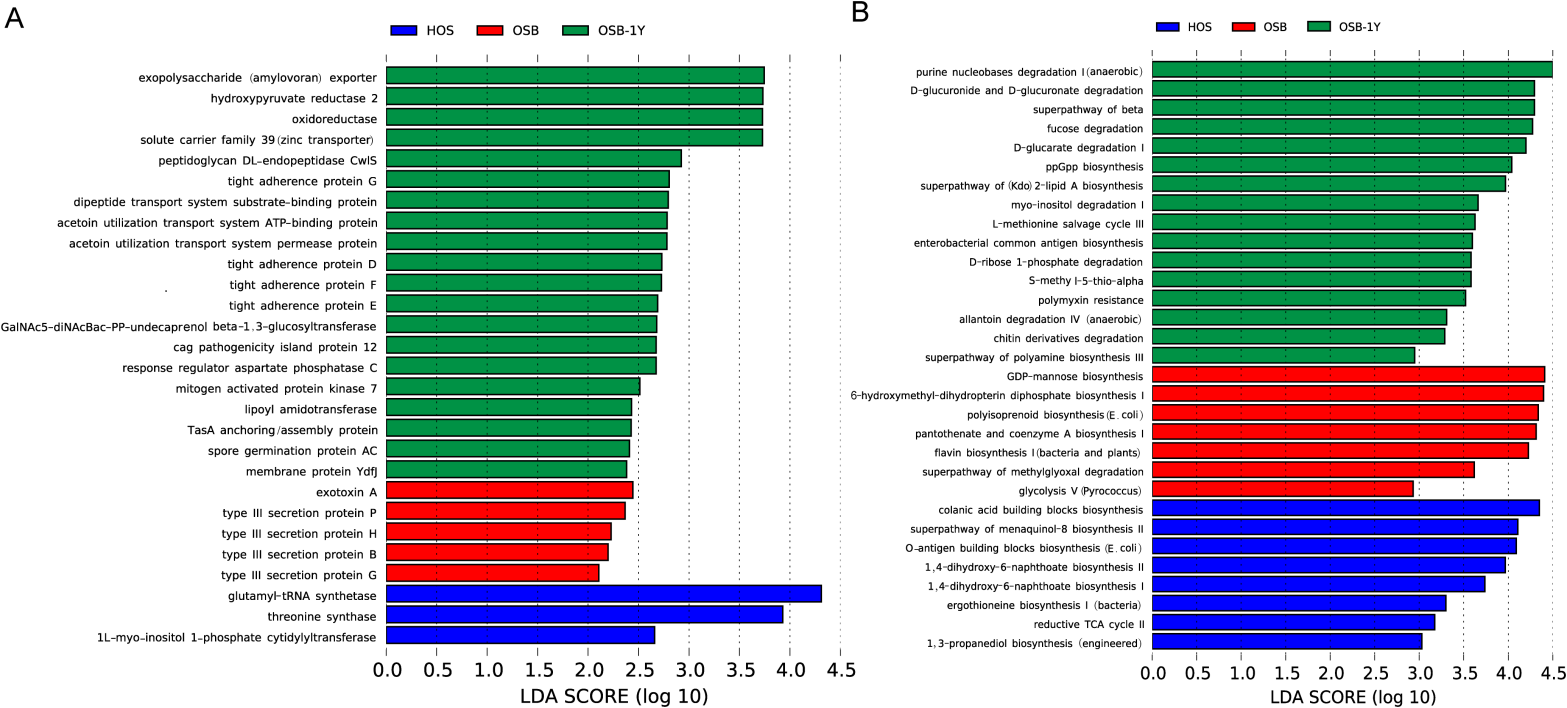
Functional changes of ocular surface microbiota. **(A)** KEGG orthology (KO) shows functional genes in the ocular surface microbiota. **(B)** Pathway is the analysis of metabolic pathways of differential genes. LDA, linear discriminant analysis. LDA score > 3.0.

PICRUSt2 was used to conduct computational inference of microbial functional potential based on 16S rRNA sequencing data. It should be noted that such predicted functional profiles are in silico exploratory results, rather than direct *in situ* functional measurements. LEfSe analysis of predicted pathways (**Figure 8A**) indicated that glutamyl-tRNA synthetase and threonine synthase pathways were relatively enriched in the HOS group. In the OSB group, four type III secretion system proteins were predicted to be increased. In the OSB_1Y group, four tight adherence-associated proteins showed predicted upregulation. PICRUSt further inferred enrichment of the polyamine biosynthesis III pathway in the OSB group (**Figure 8B**); while polyamine metabolism is known to participate in cell growth and differentiation, this computational finding does not confirm functional activity *in situ*. O-antigen–related pathways tended to be enriched in the HOS group.

## 4 Discussion and conclusions

The present study included a heterogeneous cohort with mixed chemical and thermal OSBs. While burn etiology may influence ocular surface microenvironment and microbiota composition, the small sample size precluded formal stratified or sensitivity analyses; findings should be interpreted with this heterogeneity in mind. Notably, patients received standard clinical treatments throughout the one-year follow-up period, including topical antibiotics, steroids, preservative-containing lubricants, and occasional surgical interventions. These treatments may independently affect the ocular surface microbiota and should be considered when interpreting the observed microbial shifts (Park et al., 2026). OSBs are serious injuries that cause corneal opacity and limbal stem cell deficiencies with a poor prognosis (Bizrah et al., 2019). Damage to the ocular surface may disrupt the microbial niche and lead to compositional and functional alterations.

The present study investigated the acute and long-term influence of OSB on the ocular surface microbiota and explored potential observed associations between clinical parameters and the microbial community. According to previous studies (Eslani et al., 2014), ocular surface chemical burns can cause inflammation, corneal infections, and even eyeball rupture three to seven days after injury. The most frequent causes of infections in OSB are *Pseudomonas aeruginosa* and *Staphylococcus aureus*. *Pseudomonas* and *Acinetobacter* infections are prevalent in patients with associated non-ocular diseases, particularly in burn patients requiring oxygen therapy (Nitescu et al., 2023). Our results show trends toward higher relative abundances of *Acinetobacter*, *Staphylococcus*, *Pseudomonas*, and *Sphingomonas* in the OSB group compared with HOS. These nominal observations in the acute phase of OSB in the Chinese population are consistent with earlier reports in ocular infections.

We observed nominal trends toward higher relative abundances of *Bacteroidota*, *Actinobacteriota*, and *Verrucomicrobiota* in OSB than in HOS, and exploratory trends toward lower relative abundances of *Firmicutes* and *Proteobacteria*. At the genus level, we observed trends toward increased relative abundances of *Parabacteroides*, *Bacillus*, *Acinetobacter*, *Corynebacterium*, *Pseudomonas*, and *Akkermansia* in the OSB group. Notably, *Parabacteroides*, *Akkermansia*, and *Muribaculaceae* are widely recognized as potential gut-associated or kit-derived contaminants in low-biomass ocular samples and cannot be confidently interpreted as authentic ocular residents; accordingly, these trends should be interpreted with extreme caution and no firm biological interpretation can be assigned. These overall patterns are directionally similar to those reported in dry eye disease (Li et al., 2019). *Corynebacterium* is commonly detected on skin and mucosal surfaces including the nose and eyes (Ozkan et al., 2017). In our study, *Corynebacterium* showed nominal trends toward increased relative abundance in the OSB group and trends toward decreased relative abundance in the OSB_1Y group. *Corynebacterium* has been linked to inflammatory responses and may contribute to immune alterations in ocular surface disease (St Leger and Caspi, 2018). Ocular surface damage and expanded pathogenic taxa may nominally alter the abundance of *Corynebacterium.* The relative abundance of *Corynebacterium* showed a similar nominal trend to those of *Parabacteroides* and *Muribaculaceae*. As noted above, *Parabacteroides* and *Muribaculaceae* are potential contaminants, and no biological interpretation can be assigned to these trends. Nominal negative associations between *Muribaculaceae* and Schirmer tear test values have been reported in a mouse dry eye model, which is directionally consistent with our nominal observations; however, given the potential for contamination, the clinical relevance of *Muribaculaceae* in the present study remains unclear and no mechanistic conclusions can be drawn.

One of the most interesting nominal observations was the association between ocular surface microbiota and clinical parameters at one year after OSB. *Alistipes*, *Lachnospiraceae NK4A136*, *Akkermansia*, and *Escherichia-Shigella* showed nominal associations with tear breakup time (BUT). Notably, *Akkermansia* and *Lachnoclostridium* are categorized as potential gut-derived or kit-derived contaminants; therefore, these possible associations should be interpreted with extreme caution and do not necessarily reflect authentic ocular microbial behavior. Shorter BUT indicates tear film instability and mucin deficiency, suggesting injury or loss of conjunctival goblet cells. The nominally lower abundance of these genera in the OSB_1Y group is consistent with nominal trends toward persistent tear film alterations often associated with dry eye after burn injury. However, because several of the genera included in this analysis are potential contaminants, these interpretations are strictly exploratory and cannot be considered biologically definitive.

Chronic ocular pain in OSB patients deserves quantitative evaluation because pain, photosensitivity, dryness, and itching reduce daily function. Severe ocular pain is associated with anxiety, depression, and reduced quality of life (Baral et al., 2026). We therefore used the SF-MPQ and PSQI to assess pain and sleep. Sleep quality, sleep duration, efficiency, disturbance, and daytime dysfunction showed nominal improvements at one year. Pain perception, especially the affective dimension, also decreased substantially. Pain and sleep scores represent sensitive nominal indicators for evaluating quality of life in OSB patients. No definitive links between these changes and specific microbial taxa can be established, as several abundant taxa may represent contamination rather than authentic ocular residents.

It is worth mentioning that the pathway showing the strongest trend toward upregulation in the HOS group was glutamyl-tRNA synthetase, a key enzyme involved in protein synthesis and cellular homeostasis. Glutamyl-tRNA synthetase catalyzes the aminoacylation of tRNA and is essential for maintaining normal microbial growth, metabolic stability, and community function (Ibba and Soll, 2000). The relatively high nominal expression of this pathway in healthy controls may reflect nominal trends toward intact physiological function and homeostatic stability of the normal ocular surface microbiota. In contrast, the nominal downregulation of this pathway after burn injury may indicate trends toward impaired microbial functional activity due to ocular surface damage and dysbiosis. Genes related to type III secretion system (T3SS) proteins showed nominal upregulation in the OSB group (Yang et al., 2022). *Pseudomonas aeruginosa*, a leading cause of bacterial keratitis, relies heavily on T3SS for cytotoxicity and pathogenesis, consistent with the nominal trends toward increased *Pseudomonas* in the OSB group. The major nominally upregulated functional genes in the OSB_1Y group were associated with tight junction proteins, which are essential for mucosal barrier integrity and blood–retinal barrier function (Saadane and von Lintig, 2025). We also detected nominal upregulation of the polyamine biosynthesis III pathway in the OSB group. Polyamines are closely associated with tissue growth and differentiation after injury (Moinard et al., 2005). Pathways related to O-antigen showed trends toward enrichment in the HOS group.

This study has several limitations. First, due to the low-biomass nature of the ocular surface and clinical sampling constraints, we did not include swab blanks, extraction blanks, or sequencing negative controls. Burn patients with disrupted epithelial barriers and hospital exposure may be more susceptible to environmental or reagent contamination than healthy controls. Several abundant taxa in our dataset, including *Muribaculaceae*, *Akkermansia*, *Parabacteroides*, *Lachnoclostridium*, and *Faecalibaculum*, are typical gut-associated or kit-derived contaminants. Therefore, these genera cannot be confidently interpreted as authentic ocular surface residents, and their abundances should be interpreted cautiously.

Second, we used a stool-optimized DNA extraction kit (TIANamp Stool DNA Kit), which is not specifically designed for low-biomass ocular samples. Such kits may introduce residual gut-derived microbial DNA that can be preferentially amplified, further contributing to the observed gut-related taxa. This is a well-recognized challenge in low-biomass microbiota research and represents a key methodological limitation.

Third, the study included a heterogeneous mix of chemical and thermal burns with different mechanisms, inflammatory responses, and management strategies. This heterogeneity may influence microbial composition but could not be analyzed in subgroups due to the small sample size.

Fourth, long-term topical treatments including antibiotics, steroids, preserved lubricants, and surgeries may independently alter the ocular surface microbiota and represent an important confounding factor.

Fifth, despite the longitudinal design, most statistical tests were unpaired due to incomplete follow-up and small sample size (except for the paired Wilcoxon tests for the seven matched patients), and no multiple testing correction was applied.

Sixth, detailed information regarding potential confounding factors such as systemic comorbidities, lifestyle factors, and environmental exposures was not systematically collected, which may influence ocular surface microbiota composition.

Seventh, conjunctival sac pH was not measured in this study. Despite emergency irrigation, residual alterations in ocular surface pH cannot be excluded and may represent an unmeasured confounding factor influencing microbial composition and study findings.

In conclusion, this strictly exploratory study provides nominal observational evidence that OSBs may be associated with acute and prolonged nominal alterations in the ocular surface microbiota, which show nominal trends toward associations with some clinical ocular surface parameters, pain, and sleep quality. Predictive functional profiles suggest nominal shifts in pathways related to secretion systems, barrier function, and tissue repair. However, given the low-biomass ocular environment, lack of contamination controls, use of a stool-optimized DNA extraction kit, small sample size, heterogeneous burn etiologies, treatment-related confounding, incomplete longitudinal follow-up, and lack of adjustment for multiple testing, all findings are preliminary, cannot be considered confirmatory, and must be interpreted with extreme caution. No definitive conclusions can be drawn regarding the authentic pathophysiological roles or clinical relevance of any observed microbial taxa. These observations generate exploratory hypotheses for understanding post-burn ocular surface microenvironmental changes, but no definitive biological interpretation can be established. Further controlled, adequately powered, and methodologically rigorous studies are required to validate any potential associations. 

## Data Availability

Original datasets are available in a publicly accessible repository: The original contributions presented in the study are publicly available. This data can be found here: https://ngdc.cncb.ac.cn/gsa/index (Accession number: PRJCA014805).
