# Supplementary material for: Acute and long-term effects of ocular surface burns on ocular surface microbiota: a one-year follow-up study
Source: Front Cell Infect Microbiol. 2026 Jul 3;16:1803003. doi: 10.3389/fcimb.2026.1803003 (PMC13375464; doi:10.3389/fcimb.2026.1803003)
Supplement: Supplementary file 1 [file Table1.doc]

***Supplementary Information***

**Acute and long-term effects of ocular surface burns on ocular surface microbiota: a one-year follow-up study**

**Zijing Lu^a, b^, Shouyue Xia^b^, Na Zhang ^c^, Junpeng Liu ^d^, Jihong Wang^b, d^,** ^*^

a The Affiliated Eye Hospital of Suzhou Vocational Health College, Suzhou, China

b Wuxi School of Medicine, Jiangnan University, Wuxi, China

c The First Affiliated Hospital of Guangxi University of Traditional Chinese Medicine

d Department of Ophthalmology, Affiliated Hospital of Jiangnan University, Wuxi, China

* Corresponding author.

Jihong Wang (corresponding author)

Department of Ophthalmology, Affiliated Hospital of Jiangnan University, Wuxi, China.

e-mail address: [wangjihong2025@163.com](mailto:wangjihong2025@163.com)

telephone number: (+86) 15861560633


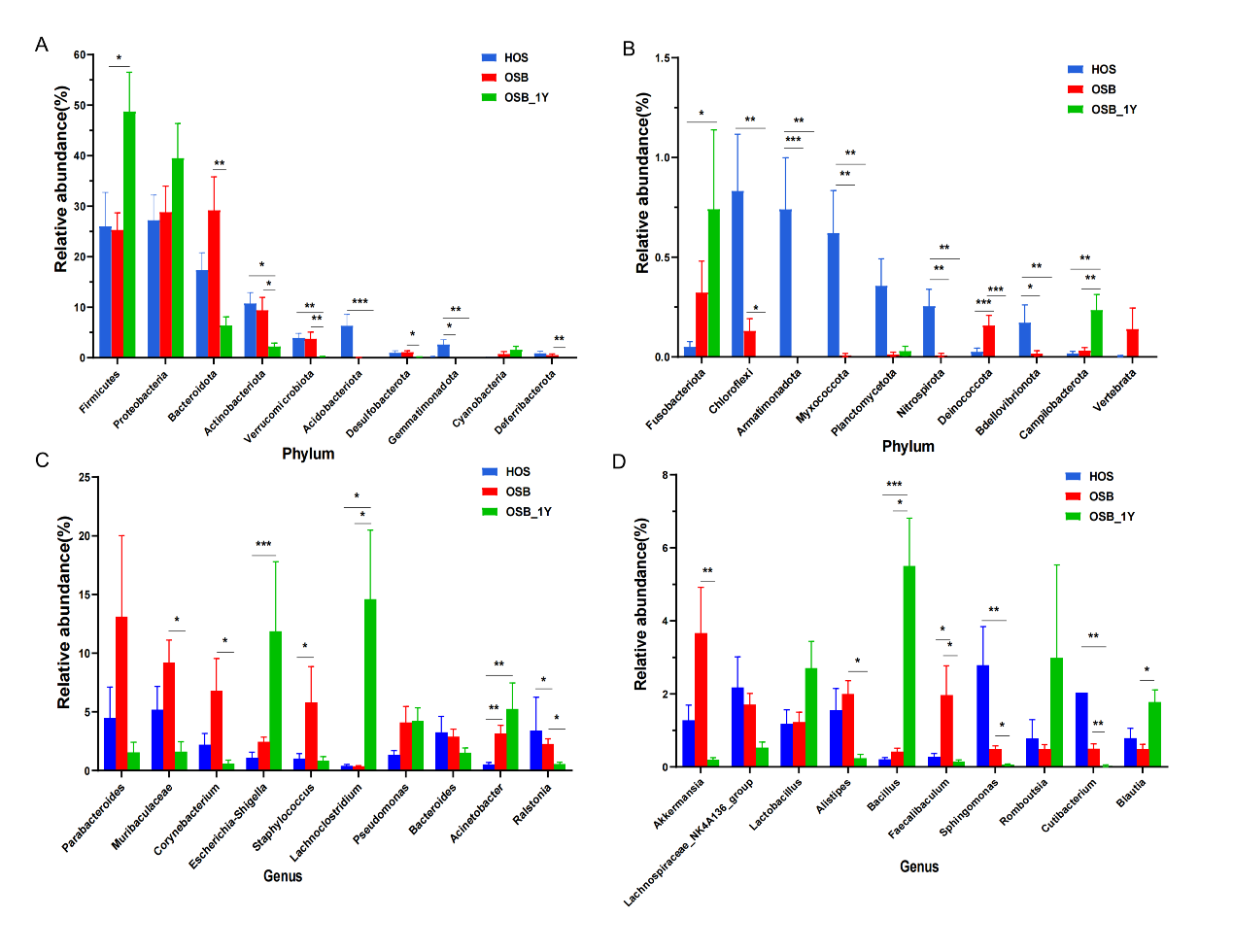


**Figure S1. The relative abundances of dominant phylum and genus among three groups.**

The top 20 ocular surface microbiota in regard to relative abundance at the phylum (A-B) and genus (C-D) level. *P<0.05, **P<0.01, ***P<0.001.


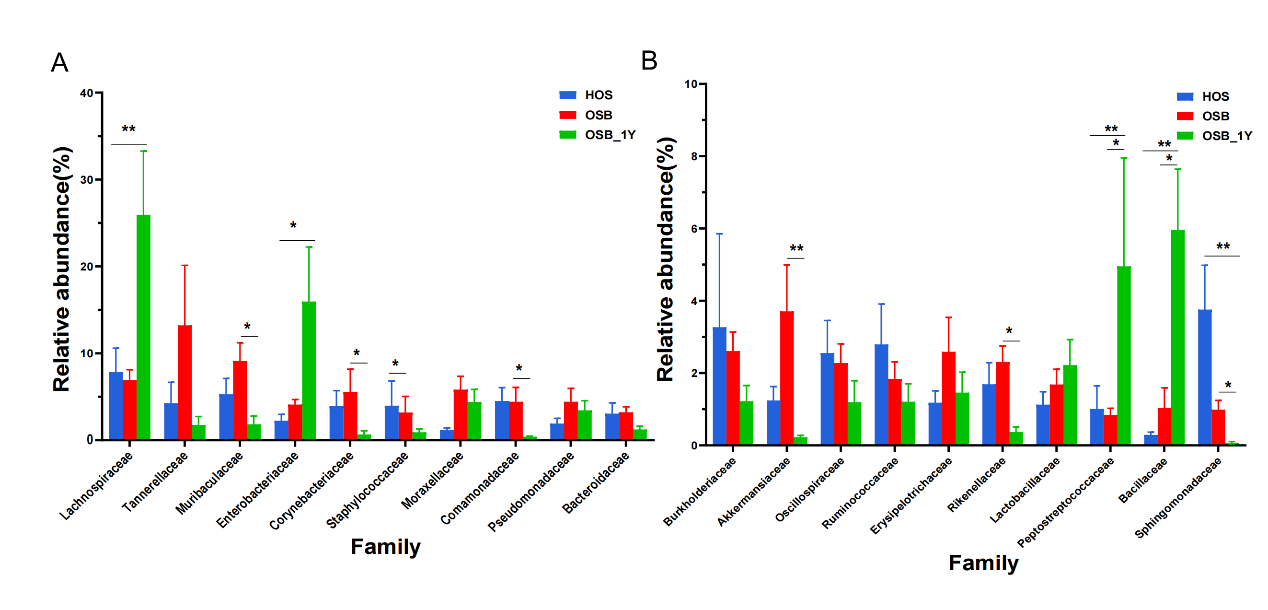


**Figure S2. The relative abundances of dominant family among three groups.**

(A-B). The top 20 ocular surface microbiota in regard to relative abundance at the family level. *P<0.05, **P<0.01, ***P<0.001

|  | **Healthy Ocular Surface (HOS)** |  | **Ocular Surface Burn (OSB)** |  |  |
| --- | --- | --- | --- | --- | --- |
|  | **N=11** |  | **N=11** | ***t*** | ***p*** |
| Gender |  |  |  | -1.491 | 0.478 |
| Male | 9(81.8%) |  | 11 |  |  |
| Female | 2(18.2%) |  | 0 |  |  |
| Age (years,mean) | 56 |  | 45 | -1.689 | 0.193 |
| Occupation |  |  |  |  |  |
| Worker | 1(9.1%) |  | 7(63.6%) |  |  |
| Farmer | 2(18.2%) |  | 1(9.1%) |  |  |
| [Office](javascript:;) [clerk](javascript:;) | 3(27.3%) |  | 2(18.2%) |  |  |
| Individual | 1(9.1%) |  | 0 |  |  |
| Retire | 4(36.4%) |  | 1(9.1%) |  |  |
| Nature |  |  |  |  |  |
| Acid | － |  | 4(36.4%) |  |  |
| Aluminum | － |  | 3(27.3%) |  |  |
| Iron | － |  | 3(27.3%) |  |  |
| Dichloromethane | － |  | 1(9.1%) |  |  |
| PSQI* Score (0-21)  (Before discharge) | 8.00±2.05 |  | 12.55±2.70 | 4.451 | **＜0.0001** |
| SF-MPQ* Score (0-60)  (Before discharge) | 5.36±1.50 |  | 19.36±4.57 | 9.659 | **＜0.0001** |
| Grade |  |  |  |  |  |
| Ⅰ | － |  | 0 |  |  |
| Ⅱ | － |  | 5(45.5%) |  |  |
| Ⅲ | － |  | 4(36.4%) |  |  |
| Ⅳ | － |  | 2(18.2%) |  |  |
| Vision |  |  |  |  |  |
| Normal vision | － |  | 3(27.3%) |  |  |
| Level Ⅱ low vision | － |  | 6(54.5%) |  |  |
| Level  II  blindness | － |  | 1(9.1%) |  |  |
| Level Ⅰ blindness | － |  | 1(9.1%) |  |  |

**Table S1. Demographic features of enrolled subjects**

*PSQI, the Pittsburgh Sleep Quality Index; SF-MPQ, the Mc-Gill Pain Questionnaire. Grade, ocular surface burn grade is based on *Dua*’s study; Visual impairment was graded according to the national standard GB‑11533‑2011: Normal vision: BCVA≥0.3;Level Ⅱlow vision: 0.1≤ BCVA < 0.3; Level  II  blindness: 0.02 ≤ BCVA < 0.05 or visual field radius < 10°; Level Ⅰ blindness: BCVA < 0.02 or visual field radius < 5. BCVA, best‑corrected visual acuity.

| **Treatment category** | **Number of patients** | **Proportion** |
| --- | --- | --- |
| Topical antibiotics | 7 | 100% |
| Topical corticosteroids | 6 | 85.7% |
| Preserved lubricants | 7 | 100% |
| Cycloplegics | 4 | 57.1% |
| IOP-lowering agents | 2 | 28.6% |
| Amniotic membrane transplantation | 2 | 28.6% |

**Table S2. Treatments received during 1-year follow-up in the OSB_1Y group (N=7)**
